# Supplementary material for: Follicular Helper and Regulatory T Cells Drive the Development of Spontaneous Epstein–Barr Virus Lymphoproliferative Disorder
Source: Cancers (Basel). 2023 Jun 3;15(11):3046. doi: 10.3390/cancers15113046 (PMC10252287; doi:10.3390/cancers15113046)
Supplement: Supplementary file 1 [file cancers-15-03046-s001.zip › cancers-2358730-supplementary/3.Supplementary-Figures_2nd-revision-revised.pptx]

## Slide 1
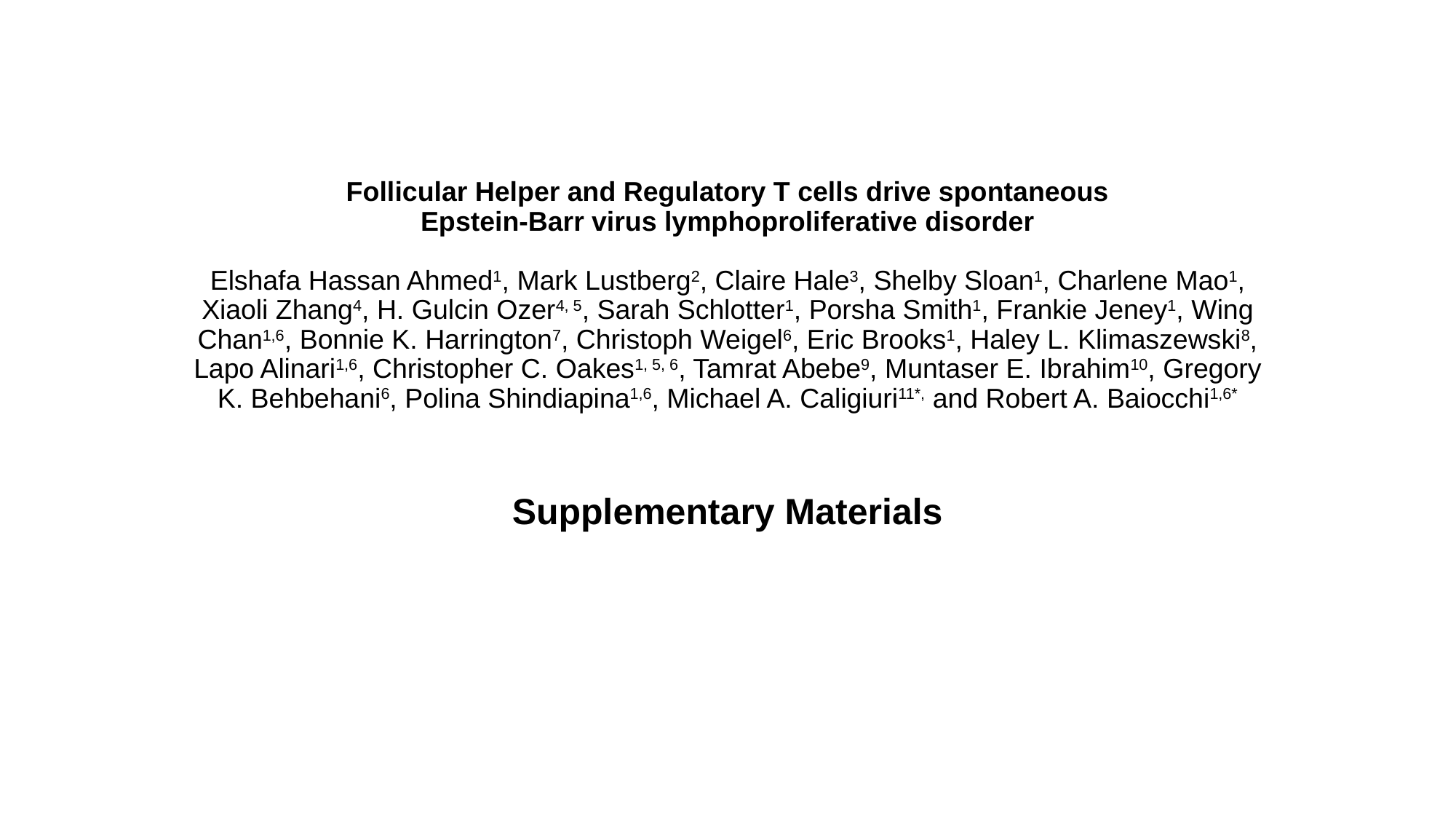

# Follicular Helper and Regulatory T cells drive spontaneous Epstein-Barr virus lymphoproliferative disorder   Elshafa Hassan Ahmed1, Mark Lustberg2, Claire Hale3, Shelby Sloan1, Charlene Mao1, Xiaoli Zhang4, H. Gulcin Ozer4, 5, Sarah Schlotter1, Porsha Smith1, Frankie Jeney1, Wing Chan1,6, Bonnie K. Harrington7, Christoph Weigel6, Eric Brooks1, Haley L. Klimaszewski8, Lapo Alinari1,6, Christopher C. Oakes1, 5, 6, Tamrat Abebe9, Muntaser E. Ibrahim10, Gregory K. Behbehani6, Polina Shindiapina1,6, Michael A. Caligiuri11*, and Robert A. Baiocchi1,6*
Supplementary Materials

## Slide 2
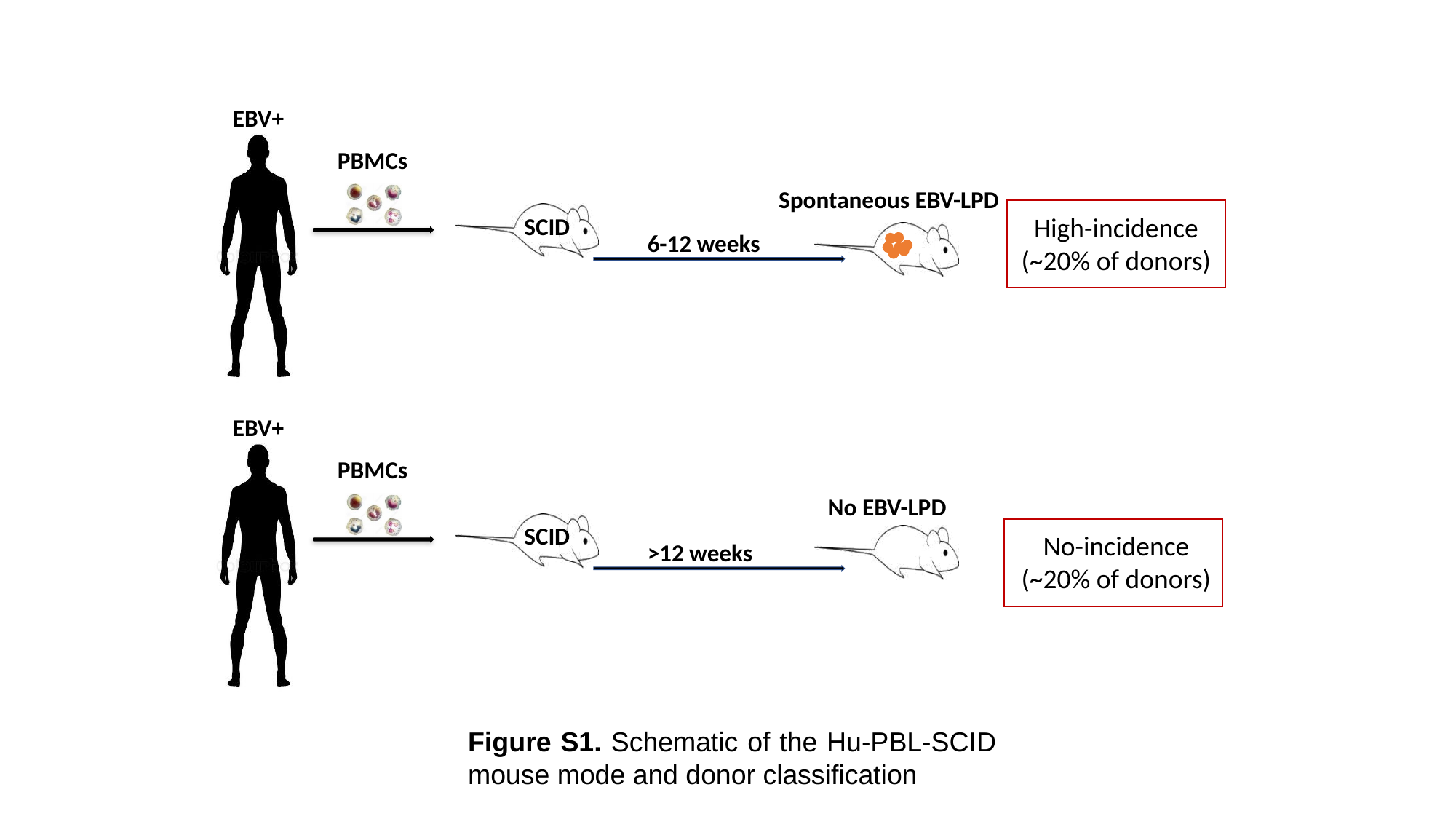

EBV+
PBMCs
Spontaneous EBV-LPD
High-incidence
(~20% of donors)
SCID
6-12 weeks
EBV+
PBMCs
No EBV-LPD
SCID
No-incidence
(~20% of donors)
>12 weeks
Figure S1. Schematic of the Hu-PBL-SCID mouse mode and donor classification

## Slide 3
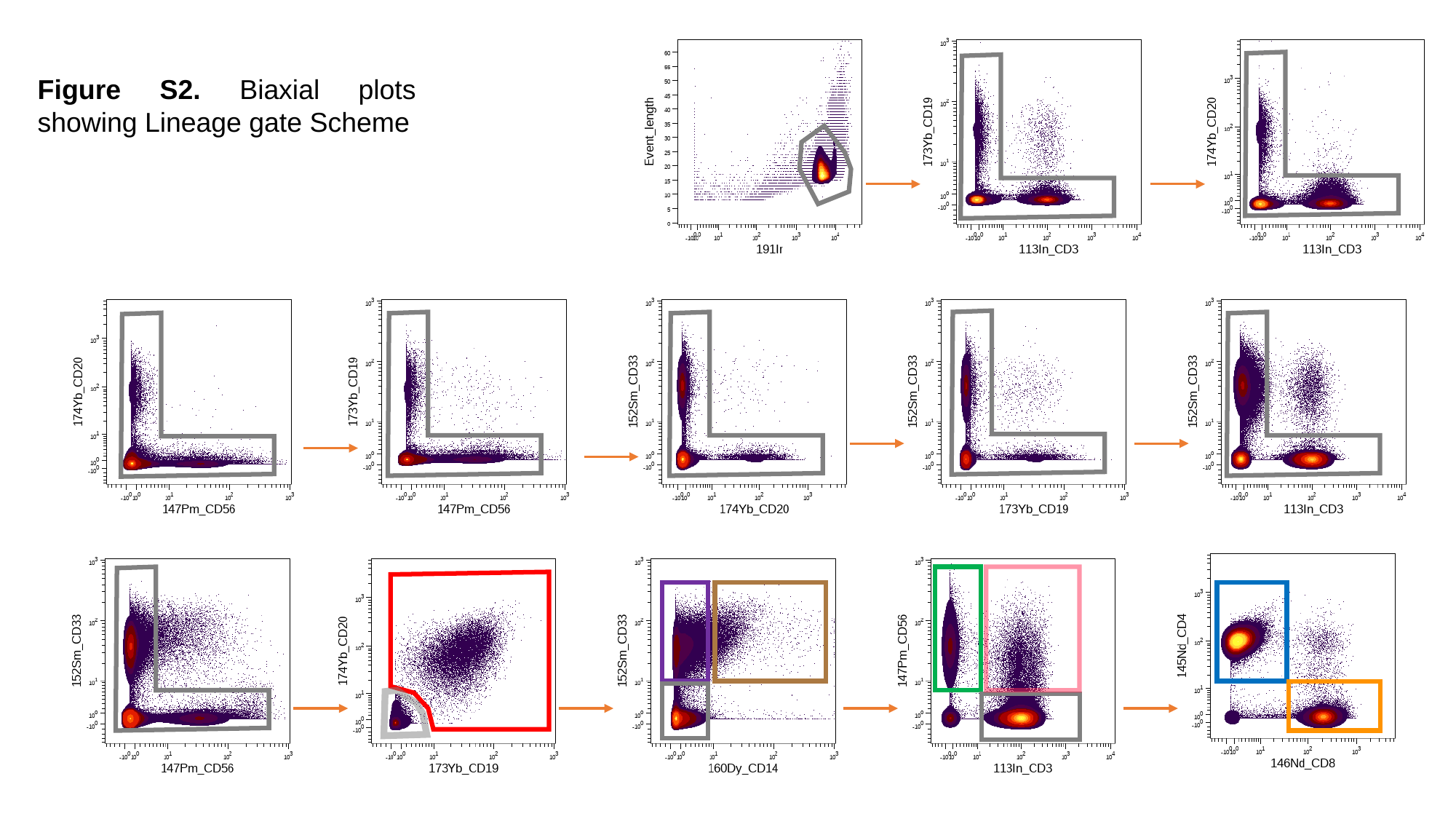

Figure S2. Biaxial plots showing Lineage gate Scheme

## Slide 4
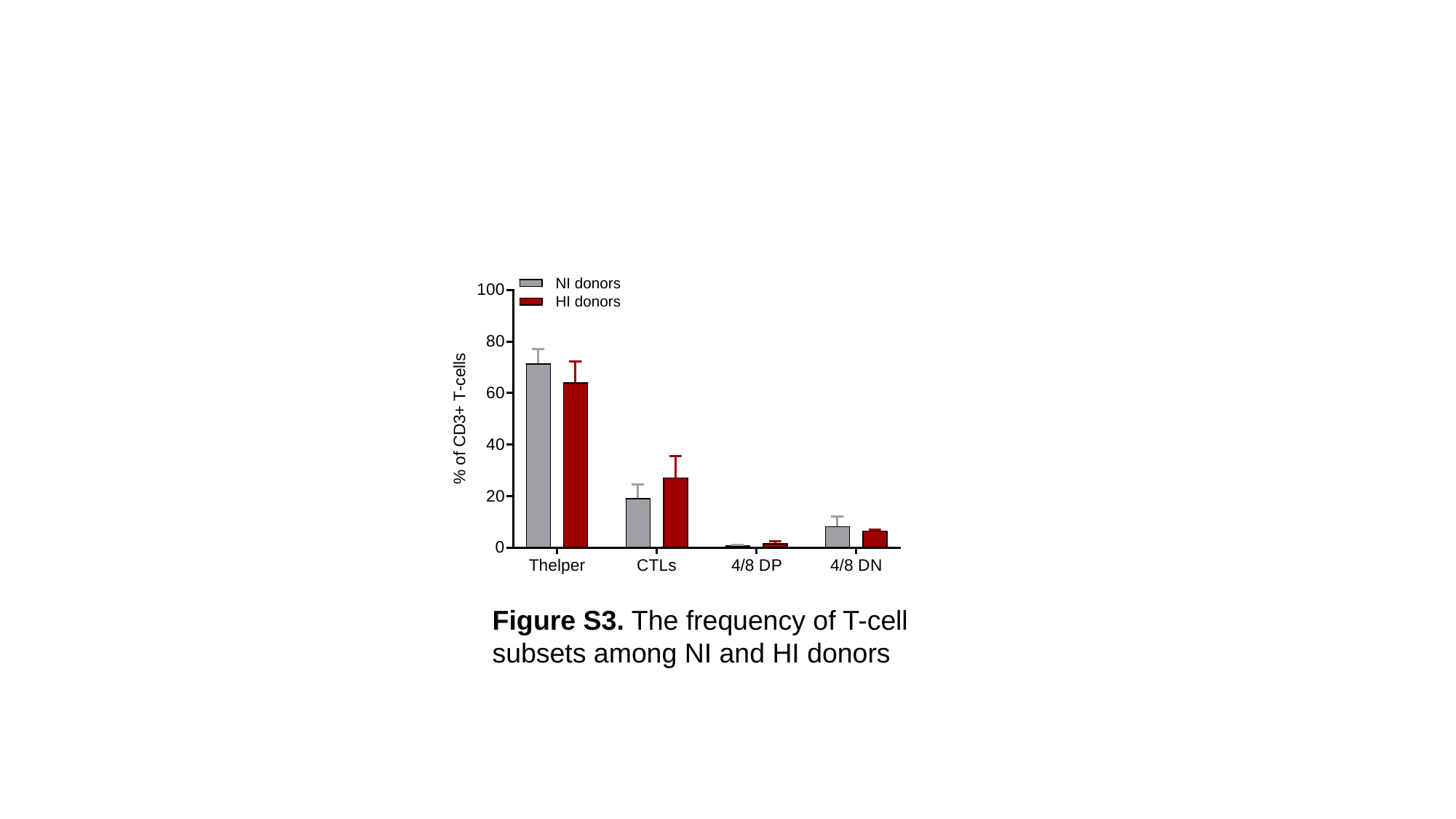

Figure S3. The frequency of T-cell subsets among NI and HI donors

## Slide 5
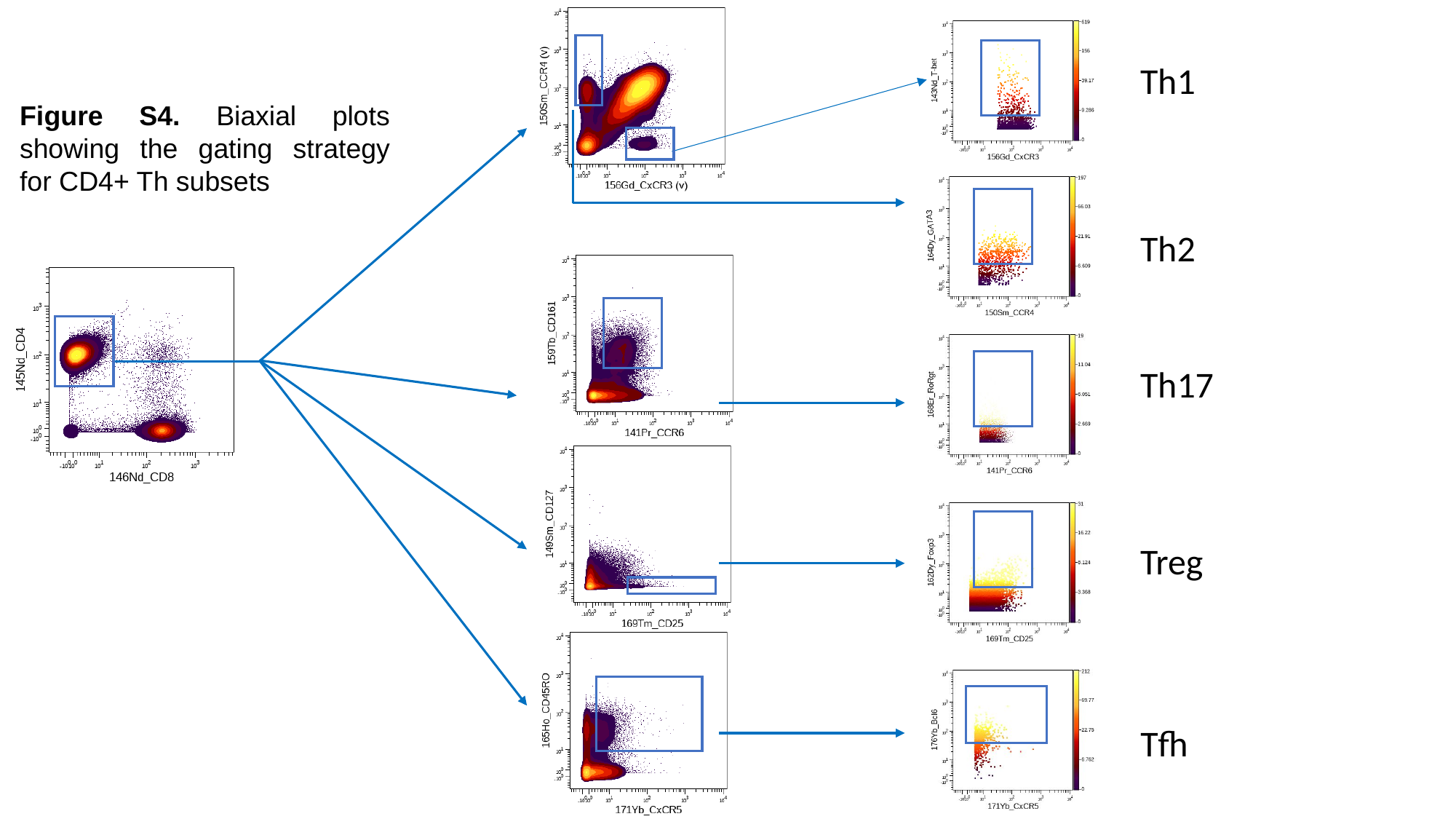

Th1
Figure S4. Biaxial plots showing the gating strategy for CD4+ Th subsets
Th2
Th17
Treg
Tfh

## Slide 6
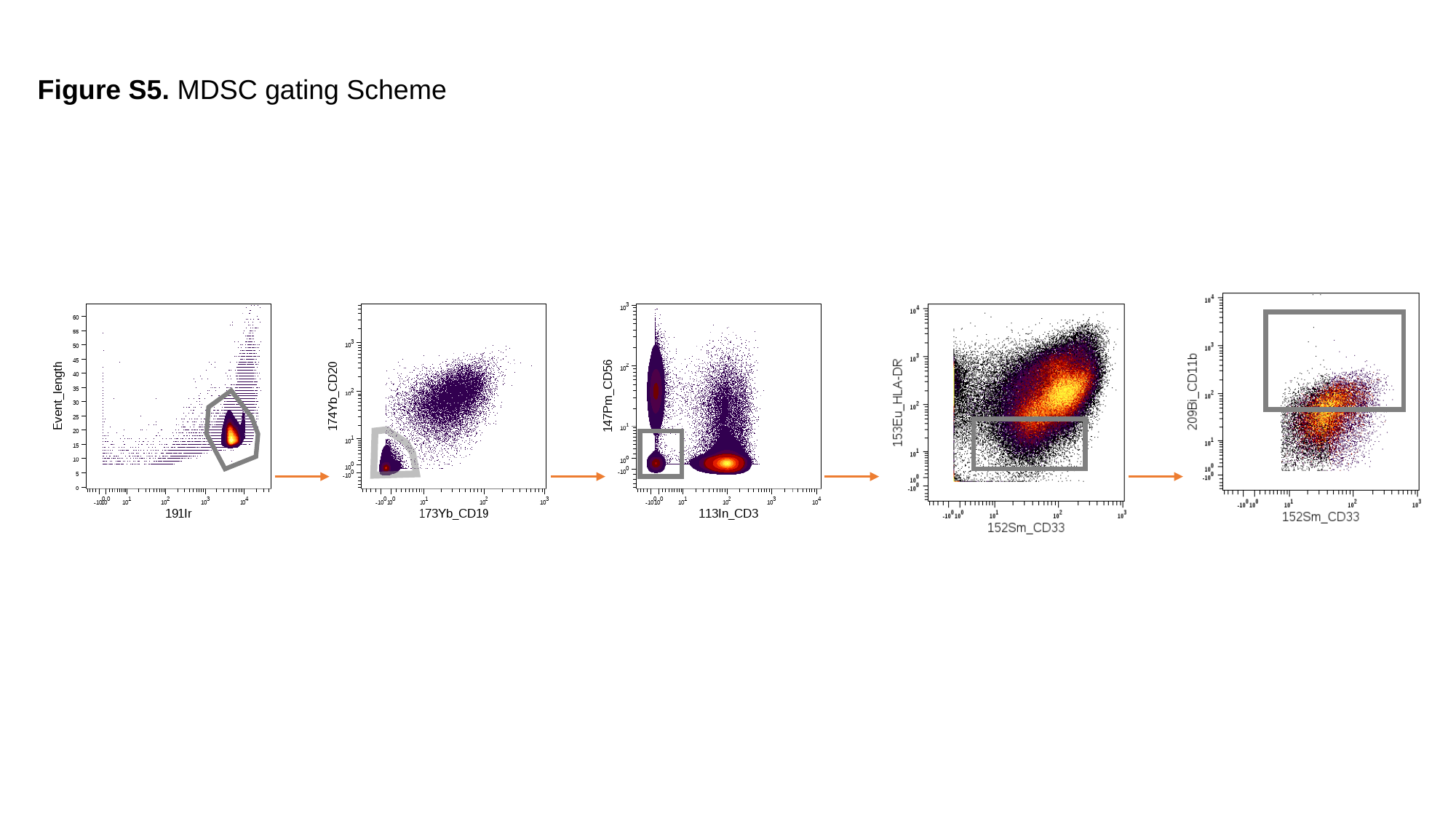

Figure S5. MDSC gating Scheme

## Slide 7
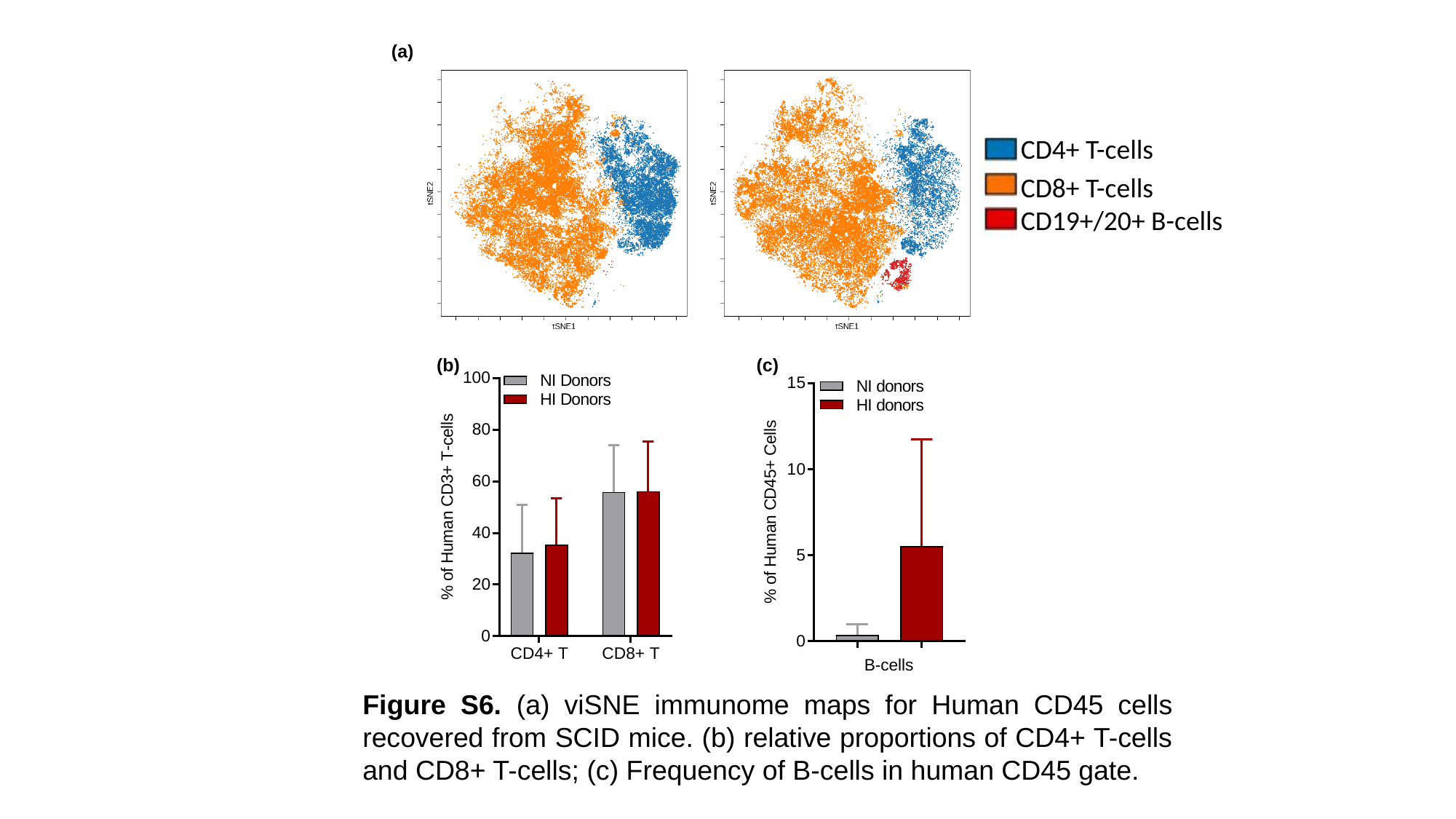

(a)
CD4+ T-cells
CD8+ T-cells
CD19+/20+ B-cells
(b)
(c)
Figure S6. (a) viSNE immunome maps for Human CD45 cells recovered from SCID mice. (b) relative proportions of CD4+ T-cells and CD8+ T-cells; (c) Frequency of B-cells in human CD45 gate.

## Slide 8
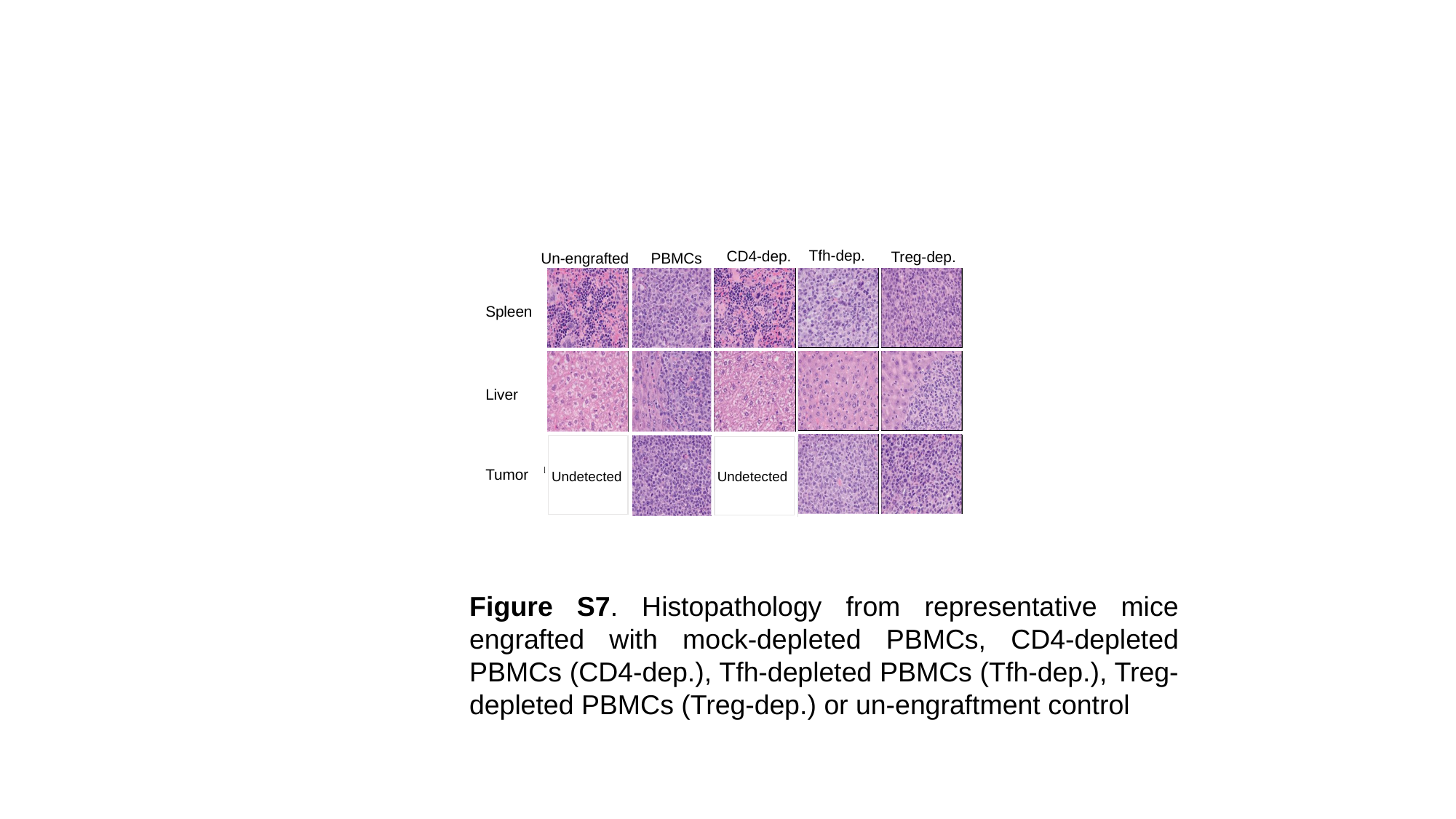

Tfh-dep.
CD4-dep.
Treg-dep.
Un-engrafted
PBMCs
Spleen
Liver
Tumor
Undetected
Undetected
Figure S7. Histopathology from representative mice engrafted with mock-depleted PBMCs, CD4-depleted PBMCs (CD4-dep.), Tfh-depleted PBMCs (Tfh-dep.), Treg-depleted PBMCs (Treg-dep.) or un-engraftment control

## Slide 9
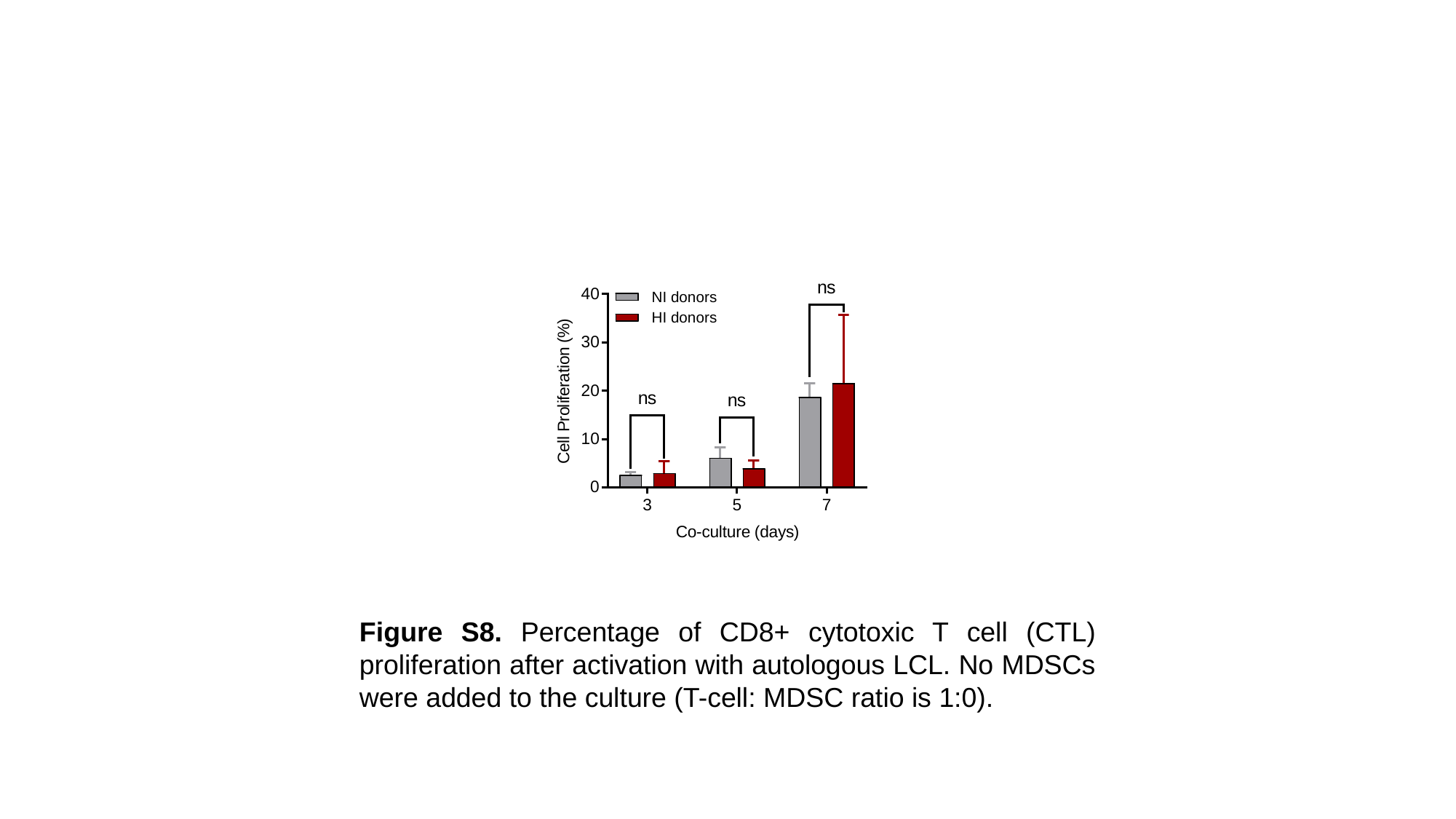

Figure S8. Percentage of CD8+ cytotoxic T cell (CTL) proliferation after activation with autologous LCL. No MDSCs were added to the culture (T-cell: MDSC ratio is 1:0).
