# Supplementary material for: Follicular Helper and Regulatory T Cells Drive the Development of Spontaneous Epstein–Barr Virus Lymphoproliferative Disorder
Source: Cancers (Basel). 2023 Jun 3;15(11):3046. doi: 10.3390/cancers15113046 (PMC10252287; doi:10.3390/cancers15113046)
Supplement: Supplementary file 1 [file cancers-15-03046-s001.zip › cancers-2358730-supplementary/3.Supplementary-Tables_2nd-revision.pdf]

Table S1. Characteristics of the Study Cohort.

LPD classification	Donor ID	Gender	% of mice with EBV-LPD	Latency time to EBV-LPD
High-Incidence	HI-1	Male	100	8 weeks
	HI-2	Male	100	6-8 weeks
	HI-3	Male	100	6-8 weeks
	HI-4	Female	100	6-8 weeks
	HI-5	Female	100	6-8 weeks
No-Incidence	NI-1	Male	0	n/a
	NI-2	Female	0	n/a
	NI-3	Female	0	n/a
	NI-4	Male	0	n/a

Table S2. List of antibodies for mass cytometry analysis.

Ab	Metal	Clone	Company	Amount per 100 μl
CD3	113In	HIR2	Biolegend	1
CD45	115In	HI30	Biolegend	1
IFNγ	139La	B27	Biolegend	1
cPARP	140Ce	F21-852	BD Pharmingen	1
CCR6	141Pr	G034E3	Biolegend	1
CD107a	142Nd	H4A3	Biolegend	1
T-bet	143Nd	eBio4B10	eBioscience	1

CD94	144Nd	HP-3D9	BD Biosciences	1
CD4	145Nd	RPA-T4	Biolegend	1
CD8	146Nd	RPA-T8	Biolegend	1
CD56	147Sm	NCAM16.2	BD Biosciences	1
CD154 (CD40L)	148Nd	24-31	Biolegend	1
CD127	149Sm	A019D5	Biolegend	1
CCR4	150Nd	205410	R&D systems	1
ICOS	151EU	DX29	BD Biosciences	1
CD33	152Sm	P67.6	Biolegend	1
HLA-DR	153Eu	L243	Biolegend	1
CD69	154Sm	FN50	Biolegend	1
PD-1	155Gd	EH12.2H7	Biolegend	1
CXCR3	156Gd	G025H7	Fluidigm	1
CD62L	157Gd	DREG-56	Biolegend	1
CD27	158Gd	L128	Fluidigm	1
CXCR5	159Tb	J252D4	Biolegend	1
CD14	160Gd	M5E2	Biolegend	1
CD16	161Dy	3G8	Biolegend	1
Foxp3	162Dy	PCH101	Fluidigm	1
GATA3	163Dy	16E14A23	Biolegend	1
GITR	164Dy	108-17	Biolegend	1
CD45RO	165Ho	UCHL1	Biolegend	1

NKG2D	166Er	1D11	Biolegend	1.5
CCR7	167Er	G043H7	Fluidigm	1
RORgt	168Er	600214	Fluidigm	1
CD25	169Tm	2A3	BD Biosciences	1
CTLA-4	170Er	14D3	eBioscience	1
GRZB	171Yb	QA16A02	Biolegend	1
PD-L1	172Yb	24F.10C12	Fluidigm	1
CD19	173Yb	H1B19	BD Biosciences	1
CD15	174Yb	W6D3	Biolegend	1
CD20	175Lu	2H7	Biolegend	1
Bcl6	176Yb	IG191E/A8	Biolegend	1
CD11b	209Bi	ICRF44	Biolegend	1

Table S3. Cells number for mouse engraftment in the Th subset depletion experiment.

Donor	% of CD4+ T in CD45	Abs CD4 Depleted PBMC Engrafted	% of Tfh in CD45	Abs Tfh Depleted PBMC Engrafted	% of Treg in CD45	Abs Treg Depleted PBMC Engrafted
HI-1	23.9%	$3.80x10^7$	3.84%	$4.80x10^7$	2.64%	$4.86x10^7$
HI-2	19.5%	$4.0x10^7$	4.0%	$4.80x10^7$	3.5%	$4.82x10^7$
HI-3	29.4%	$3.53x10^7$	2.1%	$4.89x10^7$	3.3%	$4.83x10^7$
